# Simulated warming enhances biological invasion of *Solidago canadensis* and *Bidens frondosa* by increasing reproductive investment and altering flowering phenology pattern

**DOI:** 10.1038/s41598-018-34218-9

**Published:** 2018-10-30

**Authors:** Yusong Cao, Yi’an Xiao, Sisi Zhang, Wenhai Hu

**Affiliations:** 1 School of Life Sciences, Jinggangshan University, Ji’an, Jiangxi Province 343009 People’s Republic of China; 2 Key Laboratory for Biodiversity Science and Ecological Engineering, Ji’an, Jiangxi Province 343009 People’s Republic of China

## Abstract

Phenological and reproductive shifts of plants due to climate change may have important influences on population dynamics. Climate change may also affect invasive species by changing their phenology and reproduction, but few studies have explored this possibility. Here, we investigated the impact of climate change on the phenology, reproduction and invasion potential of two alien *Solidago canadensis* and *Bidens frondosa* and one native weed, *Pterocypsela laciniata*, all of which are in the Asteraceae family. The three species responded to simulated climate change by increasing reproductive investments and root/leaf ratio, prolonging flowering duration, and while the two alien species also displayed a mass-flowering pattern. Moreover, our experimental results indicated that the alien invasive species may have greater phenological plasticity in response to simulated warming than that of the native species (*P*. *laciniata*). As such, climate change may enhance the invasion and accelerate the invasive process of these alien plant species.

## Introduction

Global mean temperature is predicted to increase 1.4–5.8 °C by 2100 as a result of growing greenhouse gases (GHG) concentrations in the atmosphere^1–3^. Climate change has already altered the population dynamics of species and their geographic ranges^4^ 4, and along with biological invasions is a key driver affecting global biodiversity^5–7^. However, the effects of climate change and biological invasion on biodiversity are usually considered separately^4,6^. Moreover, the mechanisms by which climate change influence the specific influences of climate change on phenology and reproduction of invasive plants are poorly understood^8^.

Reproduction is a fundamental challenge for invaders when they are introduced into a new habitat. Thus, reproductive traits in general become important determinants of invasion^9^. Some reproductive traits, such as reproductive allocation and clonality, are positively associated with invasion^10^. However, the role of many other traits in invasion success, including seed dispersal patterns and flowering phenology, are less well known.

Most observations of climate-change responses have involved alterations of species’ phenologies^11^ which can have major influences on plant productivity^12^ and competition among species^13–15^. Evidence suggests that many plant species have advanced the timing of their phenological events in response to warming temperatures, and that the response modes of plant phenology to temperature are often nonlinear^16^. In southern Wisconsin, 18 of 55 species advanced spring events, whereas the others showed no change in timing in the 1980s and 1990s^11,17^. Earlier phenological activity increased the probability that developing fruit would reach full maturity^15^. Munson and Sher^18^ found that the flowering date of rare species in the Southern Rocky Mountains in Colorado has advanced 3.1 days every decade since the late 1800s, which indicated that the large shifts in plant phenology was related to climate. Moreover, flowering phenology directly influences pollination and the density of flowering individuals^19^, both of which directly impact plant reproductive success. Some successful invaders generally displayed earlier flowering or longer blooming periods, though several studies showed no significant differences in flowering phenology between native and non-native plants^20^. Other studies clearly showed that it is advantageous for an invasive species to flower for longer than a native plant^21,22^. However, the effects of climate change on the phenology of invasive plants is still unclear.

Lots of references showed that climate change has induced different responses of plant in flowering onset. Simulated warming leads to a significantly earlier onset of flowering in *Silene acaulis*^23^, *Hibbertia hirsute*^24^, and *Gentiana formosa*^25^. Temperature increase causes *Arabidopsis halleri* to advance flowering onset day and to shorten flowering duration, even leading to the loss of flowering opportunity^26^. On the other hand, flowering onset of *Aster alpinus* and *Trollius farreri* on the Tibetan plateau were significantly delayed under the warming climate^27^. Variable responses of native species in flowering phenology to climate change suggest that climate change may also affect the phenology of non-native species, which in turn would influence their invasion.

The Asteraceae are proportionally over-represented among invasive plant species worldwide^28^. Both *Solidago canadensis* and *Bidens frondosa* have become worldwide invasive herbs^26^ that have had serious ecological consequences in some countries^27^. *Pterocypsela laciniata* is a native weed growing in valleys, hillsides, forest margins, thickets, grasslands and wastelands in China. With the invasion of *S*. *canadensis* and *B*. *frondosa* in China habitat for *P*. *laciniata*, which is similar to that of the two alien species, is declining. To explore how simulated warming impacts the phenology, reproduction and invasion of *Asteraceae* plant species, *S*. *canadensis*, *B*. *frondosa* and *P*. *laciniata* were chosen as the subjects for the present study. Our main hypotheses are that climate change will enhance invasive ability of plant species by altering flowering phenology to promote reproduction and by increasing the number of offspring to increase reproductive allocation.

## Materials and Methods

### Plant species

*Solidago canadensis* L. is a perennial in the family Asteraceae widely distributed in the eastern US and Canada^29^, but is considered to be a serious invasive weed because of its strong range expansion and negative effects on native ecosystems in many countries in Europe, Asia and Oceania^29–31^.

*Bidens frondosa* L. is an annual weed native to North America that grows in wet, nutrient-rich mudsoils or muddy sand-soils on roadsides or the shores of rivers and lakes^32^, or in wastelands. This species has expanded its range throughout Europe and Asia^33^ and is considered one of the most widely distributed invasive species in China.

*Pterocypsela laciniata* (Houtt.) Shih is a perennial species in the family Asteraceae. It is native to China and mainly distributed in Shandong, Zhejiang and Jiangxi Provinces.

### Experimental design

Experiments were conducted from March to December, 2013 at the biological experiment station of Jinggangshan University, China (27°06′31″–27°07′23″ N, 115°01′08″–115°02′05″ E). The climate of this area is subtropical monsoon climate. A simulated warming treatment and a control were designed to test the effects of climate change on the flowering phenology and reproductive allocation of the three tested species. Five replicates were set up for each temperature treatment in this experiment, and 6 plants were duplicated in each replicate. A total of 60 plants were planted in 60 flowerpots (21-cm d. × 21-cm h). All the plants used in the experiment were seedlings grown from seeds collected from the plants growing in the same region. Plants with similar growth potential were transplanted into the test pots for cultivation and observation.

Temperature was increased using conical OTCs that were designed to be 2.20 m in basal diameter, 0.80 m top diameter, and 1.30 m in height. The air temperature and relative humidity (at 30 cm above the soil surface) inside and outside of the OTCs were recorded at hourly intervals by an automatic temperature and humidity recorder (EM50, Decagon Devices Inc., Pullman, U.S.A) during the study period. The OTCs significantly increased the average monthly air temperature by a mean of 1.6 °C (*t*_9_ = 9.618, *P* < 0.001), but the observed decrease in relative humidity was not significant (mean difference 0.159%; *t*_9_ = 0.325, *P* = 0.581).

The flowering phenology of each plant was observed and recorded every 3 days until the last flower withered, including onset day (the day the first flower attained anthesis), endset day (the day the last flower attained anthesis), and the numbers of flowers per plant. The flowering duration, mean flowering amplitude (number of flowers per plant per day), and the relative flowering intensity and synchrony index were calculated for each species in each treatment. The number of flowers and fruits per plant was counted carefully. Seed weights of each treatment for each species were measured. The ramets of *S*. *canadensis* were also counted after harvesting.

The synchrony index (*S*_*i*_) of a population is used for detecting flowering synchrony and specific methods according to McIntosh^34^ and is given by:

1 $${S}_{i}=\frac{1}{n-1}(\frac{1}{{f}_{i}})\sum _{j=i}^{n}{e}_{j\ne i}$$

Where *e*_*j*_ ≠ *i* is the days of individual *i* and *j* overlap in their flowering, *f*_*i*_ is the total flowering days of individual *i*, *n* is the number of individuals in the sample. Note that *S*_*i*_ can range from 0–1, where 0 means there is no overlapping of flowering while 1 means there is complete overlap.

The relative flowering intensity of a plant was defined as the ratio of the number of flowers during the peak flowering stage to the largest number of flowers produced.

The plants were harvested after the height and numbers of leaves were recorded and all ripe fruits were collected. Harvested biomass was separated into roots, stems, leaves and fruits.

The biomass of each part of the plant was determined after oven drying at 80 °C for 48 hours, after which, the biomass allocation parameters were calculated as follows:

2 $${\rm{R}}{\rm{o}}{\rm{o}}{\rm{t}}\,{\rm{m}}{\rm{a}}{\rm{s}}{\rm{s}}\,{\rm{r}}{\rm{a}}{\rm{t}}{\rm{i}}{\rm{o}}\,({\rm{R}}{\rm{M}}{\rm{R}})=\frac{{\rm{R}}{\rm{o}}{\rm{o}}{\rm{t}}\,{\rm{b}}{\rm{i}}{\rm{o}}{\rm{m}}{\rm{a}}{\rm{s}}{\rm{s}}}{{\rm{T}}{\rm{o}}{\rm{t}}{\rm{a}}{\rm{l}}\,{\rm{b}}{\rm{i}}{\rm{o}}{\rm{m}}{\rm{a}}{\rm{s}}{\rm{s}}}$$

3 $${\rm{S}}{\rm{t}}{\rm{e}}{\rm{m}}\,{\rm{m}}{\rm{a}}{\rm{s}}{\rm{s}}\,{\rm{r}}{\rm{a}}{\rm{t}}{\rm{i}}{\rm{o}}\,({\rm{S}}{\rm{M}}{\rm{R}})=\frac{{\rm{S}}{\rm{h}}{\rm{o}}{\rm{o}}{\rm{t}}\,{\rm{b}}{\rm{i}}{\rm{o}}{\rm{m}}{\rm{a}}{\rm{s}}{\rm{s}}}{{\rm{T}}{\rm{o}}{\rm{t}}{\rm{a}}{\rm{l}}\,{\rm{b}}{\rm{i}}{\rm{o}}{\rm{m}}{\rm{a}}{\rm{s}}{\rm{s}}}$$

4 $${\rm{L}}{\rm{e}}{\rm{a}}{\rm{f}}\,{\rm{m}}{\rm{a}}{\rm{s}}{\rm{s}}\,{\rm{r}}{\rm{a}}{\rm{t}}{\rm{i}}{\rm{o}}\,({\rm{L}}{\rm{M}}{\rm{R}})=\frac{{\rm{L}}{\rm{e}}{\rm{a}}{\rm{f}}\,{\rm{b}}{\rm{i}}{\rm{o}}{\rm{m}}{\rm{a}}{\rm{s}}{\rm{s}}}{{\rm{T}}{\rm{o}}{\rm{t}}{\rm{a}}{\rm{l}}\,{\rm{b}}{\rm{i}}{\rm{o}}{\rm{m}}{\rm{a}}{\rm{s}}{\rm{s}}}$$

5 $${\rm{R}}{\rm{e}}{\rm{p}}{\rm{r}}{\rm{o}}{\rm{d}}{\rm{u}}{\rm{c}}{\rm{t}}{\rm{i}}{\rm{v}}{\rm{e}}\,{\rm{a}}{\rm{l}}{\rm{l}}{\rm{o}}{\rm{c}}{\rm{a}}{\rm{t}}{\rm{i}}{\rm{o}}{\rm{n}}=\frac{{\rm{F}}{\rm{r}}{\rm{u}}{\rm{i}}{\rm{t}}\,{\rm{b}}{\rm{i}}{\rm{o}}{\rm{m}}{\rm{a}}{\rm{s}}{\rm{s}}}{{\rm{T}}{\rm{o}}{\rm{t}}{\rm{a}}{\rm{l}}\,{\rm{b}}{\rm{i}}{\rm{o}}{\rm{m}}{\rm{a}}{\rm{s}}{\rm{s}}}$$

6 $${\rm{R}}{\rm{o}}{\rm{o}}{\rm{t}}/{\rm{l}}{\rm{e}}{\rm{a}}{\rm{f}}\,{\rm{r}}{\rm{a}}{\rm{t}}{\rm{i}}{\rm{o}}\,({\rm{R}}{\rm{L}}{\rm{R}})=\frac{{\rm{R}}{\rm{o}}{\rm{o}}{\rm{t}}\,{\rm{b}}{\rm{i}}{\rm{o}}{\rm{m}}{\rm{a}}{\rm{s}}{\rm{s}}}{{\rm{L}}{\rm{e}}{\rm{a}}{\rm{f}}\,{\rm{b}}{\rm{i}}{\rm{o}}{\rm{m}}{\rm{a}}{\rm{s}}{\rm{s}}}$$

7 $${\rm{R}}{\rm{o}}{\rm{o}}{\rm{t}}/{\rm{s}}{\rm{h}}{\rm{o}}{\rm{o}}{\rm{t}}\,{\rm{r}}{\rm{a}}{\rm{t}}{\rm{i}}{\rm{o}}\,({\rm{R}}/{\rm{S}})=\frac{{\rm{R}}{\rm{o}}{\rm{o}}{\rm{t}}\,{\rm{b}}{\rm{i}}{\rm{o}}{\rm{m}}{\rm{a}}{\rm{s}}{\rm{s}}}{{\rm{T}}{\rm{o}}{\rm{t}}{\rm{a}}{\rm{l}}\,{\rm{a}}{\rm{b}}{\rm{o}}{\rm{v}}{\rm{e}}\,{\rm{g}}{\rm{r}}{\rm{o}}{\rm{u}}{\rm{n}}{\rm{d}}\,{\rm{b}}{\rm{i}}{\rm{o}}{\rm{m}}{\rm{a}}{\rm{s}}{\rm{s}}\,{\rm{o}}{\rm{f}}\,{\rm{p}}{\rm{l}}{\rm{a}}{\rm{n}}{\rm{t}}}$$

### Statistical analysis

To examine the effects of warming on phenological parameters and reproduction potential for each species, T-tests were used to evaluate significant differences between treatments and controls (OTCs and CK) at α = 0.05. The analyses were performed with SPSS 18.0 (SPSS, Inc., Chicago, IL, USA) and OriginPro 8.0 (OriginLab, Northampton, MA, USA). The values reported in this paper are “Mean ± S.E.”.

## Results

### Effects on growth

**Figure 1:**
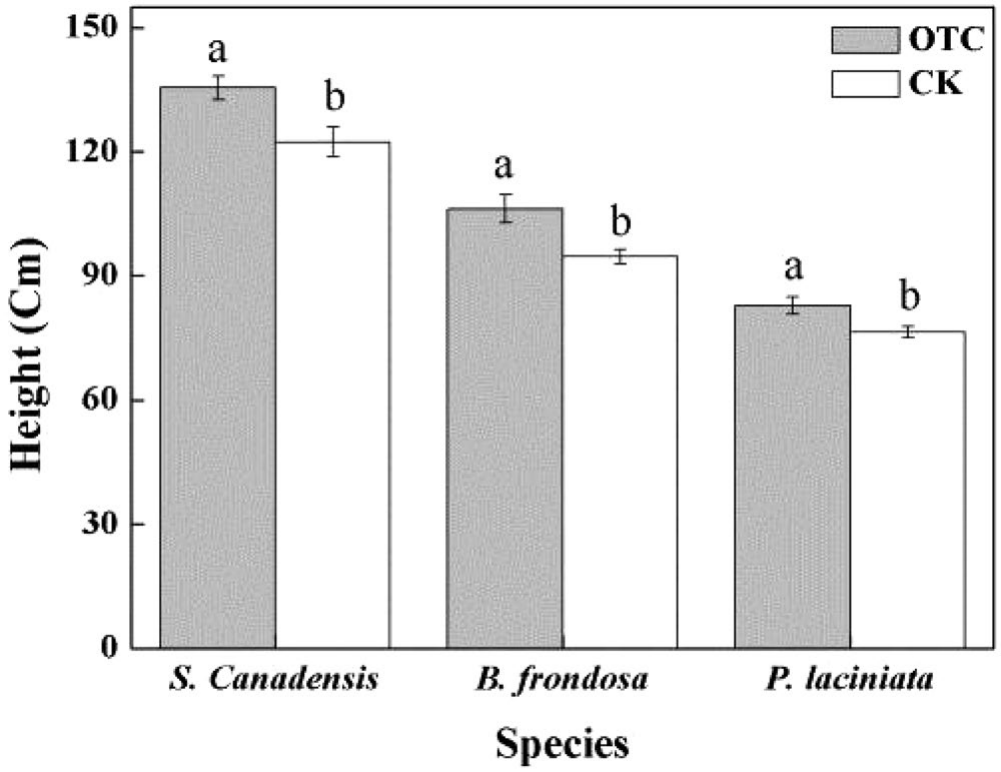
Effect of simulated warming on plant height of *S*. *canadensis*, *B*. *frondosa* and *P*. *laciniata*. Note: The values are “means ± S.E.”. Different letters indicate significant differences (*P* < 0.05).

Simulated warming significantly increased the height of each species (Fig. 1). The heights of *S*. *canadensis*, *B*. *frondosa* and *P*. *laciniata* were significantly greater in the OTC treatment (135.4 ± 2.9 cm, 106.2 ± 3.5 cm, 82.85 ± 2.0 cm, respectively) than that in the CK treatment (122.3 ± 3.6 cm, 94.6 ± 1.7 cm, 76.3 ± 1.4 cm, respectively; n = 5, *p* < 0.05).

**Figure 2:**
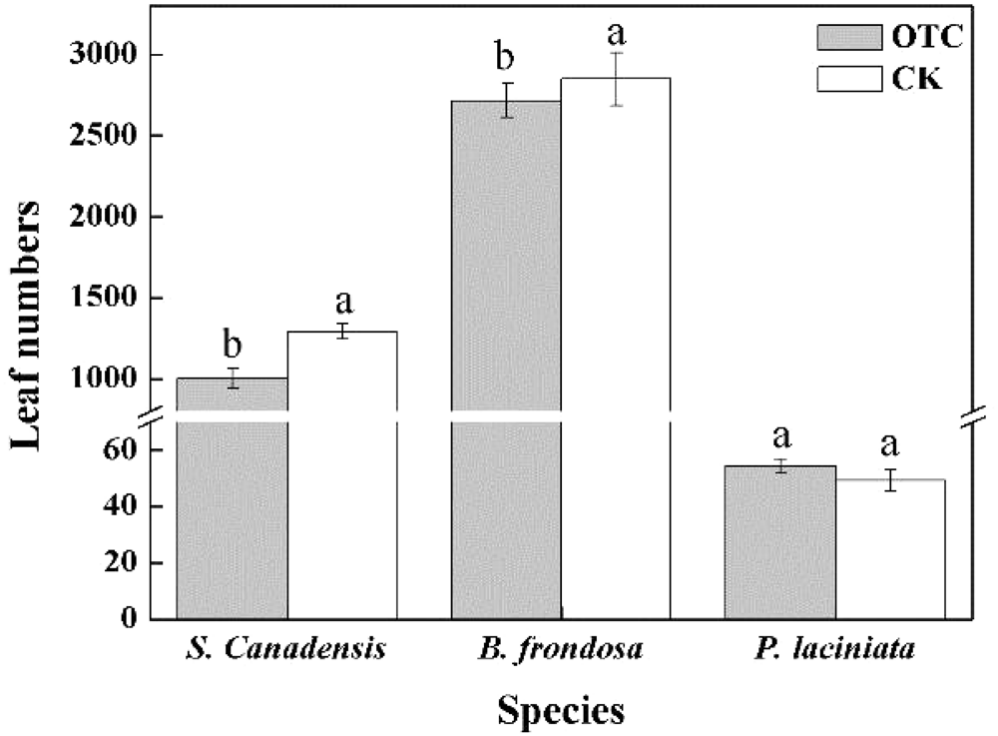
Effect of simulated warming on leaf number of *S*. *canadensis*, *B*. *frondosa* and *P*. *laciniata*.

Simulated warming decreased the leaf numbers of the two invasive plants, *S*. *canadensis* (decreased from 1291.7 ± 46.9 to 1004.2 ± 63.7) and *B*. *frondosa* (decreased from 2848.48 ± 164.4 to 2715.6 ± 105.6) (n = 5, *p* < 0.05), but had no significant effect on the leaf numbers of the native species (*P*. *laciniata*), which increased from 49.2 ± 3.2 to 54.3 ± 2.6 (Fig. 2).

### Flowering phenology

**Figure 3:**
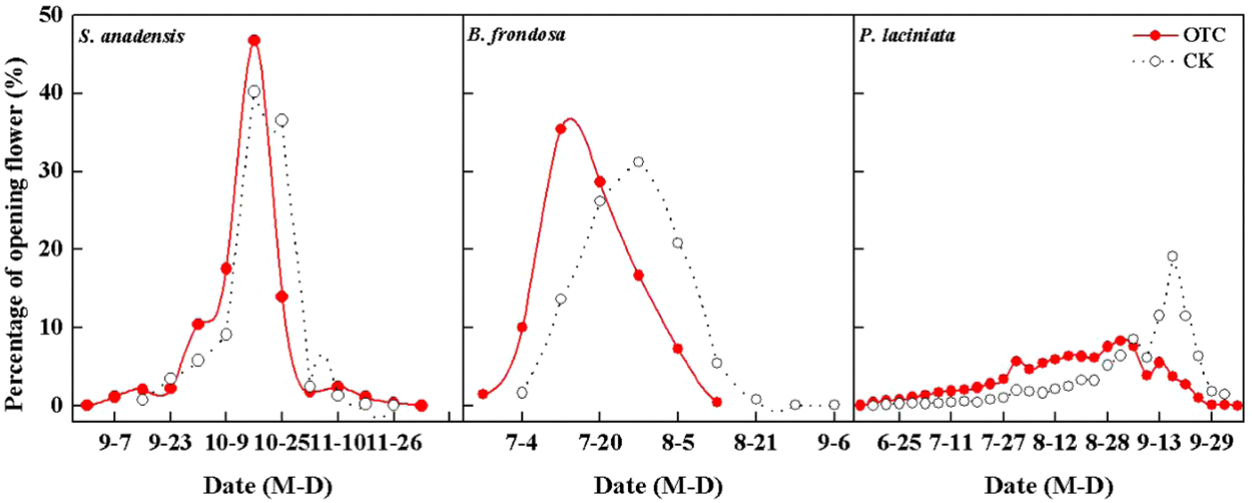
Effect of simulated warming on flowering phenology of *S*. *canadensis*, *B*. *frondosa* and *P*. *laciniata*.

The peak flowering date and endset day of *S*. *canadensis* (49.0 ± 2.7 d and 77.0 ± 1.9 d), *B*. *frondosa* (65.0 ± 1.7 d and 94.0 ± 4.3 d) and *P*. *laciniata* (77.0 ± 1.9 d and 33.0 ± 2.5 d) were significantly advanced under simulated warming treatments. Simulated warming also significantly advanced the onset day (34.0 ± 2.6 d and 9.0 ± 0.9 d) and prolonged the flowering duration (44.0 ± 2.5 d and 85.0 ± 2.8 d) of *S*. *canadensis* and *B*. *frondosa* (n = 5, *p* < 0.05) (Table 1). It was also found to increase the flowering synchrony index of the invasive species, but decrease that of the native species. Warming didn’t significantly affect the relative flowering intensity for any of the species tested (Table 1, Fig. 3).

**Table 1:** Flowering phenology index of *S*. *canadensis*, *B*. *frondosa and P*. *laciniata*.

Items	Treatment	*S. canadensis*	*B. frondosa*	*P. laciniata*
Onset day (d)	OTC	34 ± 2.645	9 ± 0.960	8 ± 1.483
	CK	38 ± 2.379	41 ± 2.873	13 ± 1.715
	P	0.016	0.001	0.139
Peak flowering date (d)	OTC	49 ± 2.684	65 ± 1.678	23 ± 1.944
	CK	52 ± 2.44	82 ± 2.250	31 ± 1.181
	P	0.014	0.008	0.041
Endset day (d)	OTC	77 ± 1.979	94 ± 4.326	33 ± 2.512
	CK	73 ± 0.759	103 ± 1.331	56 ± 1.499
	P	0.042	0.100	0.004
Duration (d)	OTC	44 ± 2.517	85 ± 2.824	26 ± 0.922
	CK	36 ± 1.102	65 ± 1.575	35 ± 1.432
	P	0.047	0.005	0.017
Flower number	OTC	5331 ± 557.78	401 ± 22.88	90 ± 4.590
	CK	5725 ± 557.34	352 ± 13.98	100 ± 2.068
	P	0.429	0.040	0.024
Flowering synchrony index	OTC	0.77 ± 0.036	0.85 ± 0.013	0.73 ± 0.022
	CK	0.72 ± 0.043	0.67 ± 0.011	0.84 ± 0.019
	P	0.014	0.001	0.020
Relative flowering intensity	OTC	0.54 ± 0.051	0.44 ± 0.064	0.53 ± 0.034
	CK	0.47 ± 0.056	0.27 ± 0.044	0.53 ± 0.035
	P	0.227	0.111	0.986

### Biomass allocation

The total biomass (64.9 ± 0.9 g · plant^−1^), leaf biomass (8.1 ± 0.1 g · plant^−1^) and shoot biomass (22.2 ± 0.6 g · plant^−1^) of *S*. *canadensis* and *B*. *frondosa* (56.8 ± 0.7, 7.4 ± 0.2 and 29.8 ± 0.5 g · plant^−1^) were significantly increased under simulated warming treatment; the root biomass of *S*. *canadensis* also increased, but not significantly. The shoot biomass of *P*. *laciniata* (0.4 ± 0.04 g · plant^−1^) was significantly increased in the warming treatment, but its root biomass decreased, and no significant difference in total biomass was displayed (Table 2).

**Table 2:** Effect of simulated warming on biomass of *S*. *canadensis*, *B*. *frondosa* and *P*. *laciniata*.

Items	Treatments	*S. canadensis*	*B. frondosa*	*P. laciniata*
Root biomass (g.plant^−1^)	OTC	29.357 ± 0.520	10.966 ± 0.371	0.37 ± 0.04
	CK	25.521 ± 0.544	10.108 ± 0.278	0.52 ± 0.04
	P	0.026	0.07	0.027
Shoot biomass (g.plant^−1^)	OTC	22.192 ± 0.582	29.779 ± 0.499	3.22 ± 0.44
	CK	20.435 ± 0.405	27.267 ± 0.463	1.92 ± 0.09
	P	0.014	0.008	0.042
Leaf biomass (g.plant^−1^)	OTC	8.115 ± 0.143	7.408 ± 0.189	0.55 ± 0.08
	CK	9.391 ± 0.307	8.425 ± 0.245	0.62 ± 0.03
	P	0.004	0.01	0.448
Flower biomass (g.plant^−1^)	OTC	5.271 ± 0.276	8.613 ± 0.159	0.34 ± 0.08
	CK	5.029 ± 0.223	7.776 ± 0.153	0.37 ± 0.04
	P	0.427	0.015	0.757
Total biomass (g.plant^−1^)	OTC	64.935 ± 0.928	56.767 ± 0.741	4.48 ± 0.60
	CK	61.376 ± 0.556	53.576 ± 0.888	3.44 ± 0.17
	P	0.04	0.034	0.158

Simulated warming significantly increased the ratio of root and shoot allocation (45.4% ± 0.4% and 34.0% ± 0.7%, n = 5, *p* < 0.05), but decreased the ratio of leaf allocation in *S*. *canadensis* (Table 3). The clonal ramets of *S*. *canadensis* increased significantly from 32.4 to 38.1 (*p* < 0.01), although its reproductive allocation didn’t increase after simulated warming. In *B*. *frondosa*, the ratio of reproductive allocation (14.8% ± 0.2%) and shoot allocation (52.7% ± 0.2%) increased significantly, while reproductive allocation of *P*. *laciniata* decreased significantly under simulated warming.

**Table 3:** Biomass allocation of *S*. *canadensis*, *B*. *frondosa* and *P*. *laciniata* by simulated warming.

Items	Treatments	*S. canadensis*	*B. frondosa*	*P. laciniata*
Root biomass ratio	OTC	45.4% ± 0.4%	19.5% ± 0.6%	8.4% ± 0.8%
	CK	43.4% ± 0.7%	19.2% ± 0.4%	15.4% ± 0.9%
	P	0.038	0.583	0.004
Shoot biomass ratio	OTC	34.0% ± 0.7%	52.7% ± 0.2%	70.9% ± 2.9%
	CK	32.8% ± 0.7%	51.0% ± 0.5%	56.0% ± 1.9%
	P	0.026	0.015	0.00.5
Leaf biomass ratio	OTC	12.6% ± 0.3%	13.0% ± 0.5%	13.3% ± 2.1%
	CK	15.6% ± 0.5%	15.9% ± 0.3%	17.9% ± 0.7%
	P	0.002	0.001	0.103
Reproductive allocation ratio	OTC	8.0% ± 0.4%	14.8% ± 0.2%	7.4% ± 0.8%
	CK	8.3% ± 0.3%	13.9% ± 0.2%	10.8% ± 0.9%
	P	0.187	0.037	0.028
Root/leaf ratio	OTC	3.722 ± 0.129	1.650 ± 0.140	0.858 ± 0.105
	CK	2.899 ± 0.113	1.231 ± 0.042	0.896 ± 0.067
	P	0.008	0.024	0.644
Root/shoot ratio	OTC	0.493 ± 0.004	0.244 ± 0.010	0.093 ± 0.109
	CK	0.473 ± 0.008	0.239 ± 0.007	0.173 ± 0.112
	P	0.058	0.600	0.004

### Seed production

Simulated warming significantly increased the seed weight for both *S*. *canadensis* and *B*. *frondosa* (0.05 ± 0.0 g and 2.8 ± 0.04 g), the germination ratio for *S*. *Canadensis* (38.8 ± 2.4%), and the seed size of *B*. *frondosa* (2.7 ± 0.04 mm) (*p* < 0.05, Table 4).

**Table 4:** Effect of simulated warming on seed of *S*. *canadensis*, *B*. *frondosa and P*. *laciniata*.

Items	Treatments	*S. canadensis*	*B. frondosa*	*P. laciniata*
1000-seed weight (g)	OTC	0.0475 ± 0.000	2.783 ± 0.044	0.37 ± 0.006
	CK	0.0465 ± 0.000	2.373 ± 0.063	0.35 ± 0.003
	P	0.021	0.005	0.053
Seed length (mm)	OTC	1.558 ± 0.009	6.609 ± 0.069	3.268 ± 0.019
	CK	1.557 ± 0.011	6.215 ± 0.057	3.260 ± 0.023
	P	0.959	0.001	0.778
Seed size (mm)	OTC	0.559 ± 0.008	2.711 ± 0.041	1.230 ± 0.019
	CK	0.548 ± 0.008	2.553 ± 0.030	1.200 ± 0.0166
	P	0.293	<0.001	0.247
Germination ratio (%)	OTC	38.8 ± 2.417	—	86.00 ± 2.608
	CK	30.0 ± 1.414		91.20 ± 1.625
	P	0.024		0.24

Note: The germination ratio of was not statistically analyzed, because there were only 3 seeds germinated in OTC but not seed germinated in CK.

## Discussion

Temperature can directly or indirectly affect plant growth and biomass production. In the present study, the results showed that simulated warming increased the accumulation of root biomass, shoot biomass and total biomass in *S*. *canadensis*, and the flower biomass and total biomass in *B*. *frondosa* (Table 3), but decreased the root biomass in the native species *P*. *laciniata* (Table 1). These results suggested that warming significantly promotes the growth of our focal invasive species but not of the noninvasive species. *S*. *canadensis* can be cloned and propagated by subterranean stem sprouts. The rhizome can accommodate clonal growth by controlling the ratio of buds per node. Our results also showed that simulated warming significantly increased the ratio of root and shoot allocation, but decreased the ratio of leaf allocation in *S*. *canadensis* (Table 3), which indicated that simulated warming may promote the clonal reproduction of *S*. *canadensis*. These responses allow the invasive species to develop dominant populations in communities and become more competitive than noninvasive species.

Plants can change their biomass allocation pattern in response to a change of environmental conditions^35^. Invasive species have previously been shown to produce more biomass than native species^36^. In the present study, simulated climate change increased the shoot biomass of both the invasive species and native species, and promoted plant height growth, increasing the size of the plant stem. Plant stem tissue is large in these species, being a location for high energy storage, which could account for the observed increase in biomass. Increased investment in shoot biomass is conducive to plant growth and can improve competitive ability. Climate change also increased the root biomass allocation and decreased the leaf biomass allocation thereby significantly increasing root/leaf ratio in *S*. *canadensis*. This pattern is likely to increase water and nutrient utilization efficiency and increase the plant’s ability to adapt to varied environmental conditions and further enhance its competitive ability and invasion.

In contrast, a non-clonal species, *B*. *frondosa* responded to the simulated warming by increasing inflorescence biomass, total biomass and root/leaf ratio. This increase in biomass allocation to reproduction organs may increase population growth and colonization of new habitats. Hence, our results from simulated climate change are consistent with the hypothesis that warming may increase the invasion of non-native species^37^. The native species, *P*. *laciniata* significantly decreased its reproductive allocation, root mass, and root/shoot ratio under climate change. These results suggest that invasive species can improve their ability to adapt to the environment and enhance invasion by optimizing resource allocation to adapt to climate change.

Flowering phenology can affect pollinators’ behavior and their sexual reproductive fitness, and further affect plant reproductive success. Plants display a variety of responses to climate change^38^. Several studies have demonstrated that within a population, early-flowering plants produce more flowers and seeds than late-flowering plants^39,40^. All three focal species significantly advanced their onset day and peak flowering day, but this resulted in a prolonged flowering season for the two invasive species and a shortened season for the native species. At the same time, our results also indicated the invasive species increased their flowering synchrony, but the native species’ decreased after warming. Flowering pattern is an important factor affecting pollination success^39^; species with high flowering synchrony and a mass-flowering pattern can be more effective in attracting pollinators^41–43^.

Although climate change could provide new habitats for invasive species, they must overcome many unfavorable factors to invade the new habitat successfully^44^. For instance, they need to get enough nutrients to complete morphogenesis, and need enough pollinators to complete the process of sexual reproduction, a process that is impacted by flowering pattern, as discussed above. Flowering phenology and pattern can be an important factor for invaders in their successful colonization in new habitat.

It has been suggested that invasive species can respond better to climate change than native species by adjusting flowering times^45^. Our results demonstrated that the invasive species, *S*. *canadensis* and *B*. *frondosa*, displayed more plasticity than the native *P*. *laciniata* do in reproductive phenology in response to simulated climate change. The invasive species shifted and prolonged its flowering pattern. This result suggests that climate change might accelerate the process of biological invasion.
